# Practice on pressure ulcer prevention among nurses in selected public hospitals, Tigray, Ethiopia

**DOI:** 10.1186/s13104-020-05049-7

**Published:** 2020-04-10

**Authors:** Hagos Berihu, Tewolde Wubayehu, Tewolde Teklu, Teklay Zeru, Hadgu Gerensea

**Affiliations:** 1 School of Nursing, College of Health Sciences and Comprehensive Specialized Hospital, Aksum University, P.O.Box: 298, Tigray, Ethiopia; 2 School of Medicine, College of Health Sciences and Comprehensive Specialized Hospital, Aksum University, Tigray, Ethiopia; 3 Departement of pharmacy, College of Health Sciences and Comprehensive Specialized Hospital, Aksum University, Tigray, Ethiopia

**Keywords:** Pressure ulcer prevention, Nurse’s pressure ulcer prevention practice

## Abstract

**Objectives:**

Pressure ulcers are localized cellular damages to the skin and underlying tissues caused by pressure, shearing and frictional force. The aim of this study is to assess practices towards pressure ulcer prevention among nurses in the Central Zone of Tigray, Ethiopia, from September 10, 2017 to June 15, 2018. This study has also identified the major barriers that hamper nurses from preventing pressure ulcers. These barriers were heavy workload, inadequate training, and lack of universal guideline and shortage of resource. 17.2% of the participants had a good practice and 82.2% of the respondents had a poor practice of pressure ulcer prevention.

**Result:**

Finding of this study showed that respondents have inadequate knowledge which may have led to their poor practice towards pressure ulcer prevention. Immediate intervention should be done on public hospitals of central Tigray to improve nurses’ practice towards pressure ulcer prevention.

## Introduction

Pressure ulcers are a type of injury that breaks down the skin and underlying tissue when an area of skin is placed under constant pressure for certain periods causing tissue ischemia, cessation of nutrition and oxygen supply to the tissues and eventually tissue necrosis [1]. Pressure ulcers are caused by a local breakdown of soft tissue as a result of compression between a bony prominence and an external surface [2, 3]. A patient with a pressure ulcer suffers from pain and discomfort, and also may have prolonged illness, delayed rehabilitation, increased hospital stay, disability and may even face death resulting from the ulcer and its complications [4, 5].

A systematic review reported that the incidence rates of pressure ulcers varied considerably by clinical setting; ranging from 0.4 to 38% in acute care, from 2.2 to 23.9% in long term care, and from zero to 17% in home care [6].

Nurses have poor knowledge regarding risk factors of pressure ulcer development [7].World Stop Pressure Ulcer Day showed that nearly 700,000 patients were affected by pressure ulcers each year and more than 2.5 million people in the USA develops pressure ulcers each year [8, 9]. Moreover, a worldwide health care budget that is spent on prevention and treatment of patients with extended hospital stays from pressure ulcer development amounts more than a billion dollars [10–13].

In Ethiopia, in Felegehiwot Referral Hospital, among 422 patients, the overall prevalence rate of pressure ulcers was 16.8%. Of this, 62%, 26.8% and 2.8% developed stage I, II and stage IV pressure ulcer, respectively [14].

Major barriers that preclude nurses to practice pressure ulcer prevention (PUP) were identified with the notable ones being a heavy workload/inadequate staff, shortage of resources and inadequate training about PUP [15, 16]. Lack of knowledge, skills, and negative attitudes towards PUP contributes significantly to the occurrence or worsening of the pressure ulcer. Pressure ulcer remains a significant and complex health problem in hospitals in terms of human suffering, tissue necrosis, pain, septicemia, loss of productivity, and financial burden [17].

There is very few published paper regarding nurses’ practice towards PUP in Ethiopia [18].

## Main text

### Study area and period

The study was conducted from September 10 to June 15, 2018, in public hospitals of Central Zone Tigray, Ethiopia.

### Study design

A cross-sectional study using a quantitative method was employed in the selected public hospitals in Central Zone, Tigray, Ethiopia.

### Source of population

All nurses working currently in public hospitals in Central Zone, Tigray, Ethiopia.

### Study population

The study population was all nurses working in the three hospitals.

### Inclusion and exclusion criteria

#### Exclusion criteria

Nurses who are severely ill and in annual leave during the data collection period were excluded.

### Sample size determination

The sample size was calculated using a single population proportion formula with consideration of the following assumptions. The proportion of the prevalence of the pressure ulcer was 16.8% which was derived from a study conducted in Ethiopia, Bahirdar Felegehiwot Referral Hospital [14]; confidence level at 95% and marginal error of 5%. The sample size was calculated as follows: the total calculated samples size was 215. Since the total population is 240, the sample size was determined using correction formula, and adding a 10% non-response rate and the final sample size was 125.

### Sampling procedure

There are three general public hospitals in Central Zone of Tigray. From these hospitals, study participants were selected using simple random sampling (lottery method). The list or frame of nurses working in all hospitals was obtained from the monthly work schedule prepared by the head nurse of each ward.

### Dependent variable

Practice of nurses towards pressure ulcer prevention.

### Independent variables

Socio-demographic factor such as

- Age
- Income
- Sex
- Knowledge of nurses
- Year of service (work experience)
- Level of education

Hospital factors such as

- Equipment
- Training guideline

Nurses’ factors such as

- Heavy work load
- Lack of time
- Job satisfaction
- Patients factor (uncooperativeness)

### Operational definitions

*Adequate knowledge of PUP* For this study, a nurse is said to have an adequate knowledge of PUP if he or she answers correctly greater than or equal to 80% of 20 pressure ulcer prevention knowledge tests.

*Inadequate knowledge of PUP* For this study, a nurse is said to have an inadequate knowledge of PUP if he or she answers correctly less than 80% of 20 pressure ulcer prevention knowledge test.

*Good practice of PUP* For this study, a nurse is said to have a good practice of PUP if he or she answers correctly greater than or equal to 80% of 20 PUP practice test.

*Poor practice of PUP* For this study, a nurse said to have a poor practice if he or she answers correctly less than 80% out 20 possible questions about their practices towards PUP

*Perceived barriers* if nurses answered “yes” or “no” with the listed barriers in the perceived barrier questionnaire.

### Data collection tool

The structured questioner for knowledge and practice of pressure ulcer was adopted from a study conducted on general hospital Lahore [19] but, socio-demographic items were modified according the socio-cultural context of the community.

### Data collection procedure

The self-administered structural questionnaire was distributed to the nurses following a request for consent from nurses and support from matrons and head nurses. Continuous follow-up and supervision were given by supervisors throughout the data collection period.

### Data quality assurance

In order to maintain the quality of the data, the data collectors received training on data collection procedure. The questioner was designed carefully, and the English version was used for the actual data collection. Before the actual data collection, the questioner was checked for clarity, comprehensiveness, and content validity by pretesting on 10% of the sample size (11 nurses working in Shire Suhul hospital, Ethiopia). Finally, possible adjustments or modifications were made on the tools by the group members. Then the collected data was cross-checked for completeness and consistency each day by the principal investigators.

### Data process and analysis

The data was entered, and analysis by using the statistical package for social science (SPSS) version 22 statistical software. The association between the dependent and independent variable was analyzed first in the binary logistic regression model. In the second step, predictor variable having a p-value < 0.3 was retained and entered to the multivariate logistic regression analysis. A p-value 0.05 was considered as cut off point for predictors to be significantly associated with outcome variables (CI-95%).

### Results

#### Socio-demographic characteristics of the nurses

A total of 125 professional nurses were invited to participate in the study, and the response rate was 97.6% of which the majority of the respondents (55.7%) were females. The mean ages of the participants were 29 with the minimum age being 22 and maximum 45 years. However, the majority of them (69.7%) were within the age group of 2029 years. More than half (53.3%) were single whereas 45.1% of them were married and 1.6% of respondents were widowed and divorced. Regarding the educational status, the majority of them (79.5%) were first-degree holders, while one-fifth of them (20.5%) were diploma holders.

#### Knowledge of nurses on pressure ulcer

The overall nurses’ knowledge of PUP was 82.75% which indicated that nurses’ knowledge regarding pressure ulcer prevention was at the high level. From the study participants, 29.5% (n = 36) had adequate knowledge about PUP (Table 1).

**Table 1:** Frequency distribution of nurses’ knowledge score towards pressure ulcer prevention practice in public hospitals in central zone Tigray 2018 (N = 122)

Nurses’ knowledge score of pressure ulcer prevention				
Variables	Correct		Incorrect	
	N	%	N	%
Risk factors for PU development				
Risk factors for development of pressure ulcers are immobility, incontinence, impaired nutrition, and altered level of consciousness	113	92.6	9	7.4
Hot water and soap may dry the skin and increase the risk for pressure ulcers	61	50.0	61	50.0
It is important to massage bony prominences	70	57.4	52	35.2
Risk assessment for PU development				
All hospitalized individuals at risk for pressure ulcers should have a systematic skin inspection at least daily and those in long-term care at least once a week	82	67.2	40	32.8
The first sign of pressure ulcer development is open sore	54	44.3	68	55.7
All individuals should be assessed on admission to a hospital for risk of pressure ulcer development	79	64.8	43	35.3
A turning schedule should be written and placed at the bedside	83	68.0	39	31.9
A Braden scale is risk assessment tool used for assessing pressure ulcer	77	63.1	45	36.8
Skin care to prevent PU				
Patient skin should be clean and dry to prevent risk of pressure ulcer development	100	82.0	22	18.0
Persons confined to bed should be repositioned every 3 h	71	58.2	51	40.8
Heel ulcer is prevented by putting pillow under the patient’s leg	91	74.6	31	25.4
A low-humidity environment may predispose a person to pressure ulcers	65	53.3	57	46.7
For persons who have incontinence, skin cleaning should occur at the time of soiling and at routine intervals	84	68.9	38	31.2
Nutrition to maintain healthy skin				
Adequate dietary intake of protein and calories should be maintained during illness	98	80.3	24	19.7
Vitamin C and E are important to maintain skin integrity	97	79.5	25	20.5
Serum albumin test is the appropriate laboratory test for nutritional assessment of pressure ulcer patient	72	59.0	50	41.0
Mechanical loading management				
The head of the bed should be maintained at the lowest degree of elevation no higher than a 30° angle consistent with medical conditions	84	68.9	38	31.2
A person who cannot move him or herself should be repositioned every 2 h while sitting in a chair.	93	76.2	29	23.8
Friction may occur when moving a person up in bed	84	68.9	38	31.2
Educational program				
Educational programs may reduce the incidence of PUs	97	79.5	25	20.5

#### Nurses’ practice towards pressure ulcer prevention

From the study, 17.2% (n = 21) of respondents had a good practice PUP and 82.2% (n = 101) of the respondents had a poor practice of PUP.

According to this finding, the nurses’ practice test scores showed that 50.33% of nurses always make pressure ulcer prevention practices, 36.46% make pressure ulcer prevention practice sometimes and 15.03% never make pressure ulcer prevention practice (Table 2).

**Table 2:** Frequency distribution of nurses’ practice score about pressure ulcer prevention in public hospitals in central zone Tigray, 2018 (N = 122)

Variables	Practice score		
	Always (%)	Sometimes (%)	Never (%)
Observing how other nurses assess	64 (52.5)	50 (40.0)	8 (6.6)
Identifying PU contributing factors	72 (59.0)	42 (34.4)	8 (6.6)
Avoid dragging patients	68 (55.7)	42 (34.4)	12 (9.8)
Avoid massaging bony prominences	70 (57.4)	38 (31.1)	4 (11.5)
Performing skin assessment	57 (46.7)	52 (42.6)	13 (10.7)
Using assessment scale	44 (36.1)	68 (49.2)	10 (14.9)
Documenting all data related to PU	58 (47.5)	50 (41.0)	14 (11.5)
Assess and provide pain management	70 (57.4)	36 (29.5)	16 (13.1)
Providing skin care	60 (49.2)	49 (40.2)	13(10.7)
Placing pillow under patient leg	66 (54.1)	47 (38.5)	9 (7.4)
Using or advancing care givers to use creams	57 (46.7)	40 (32.8)	25 (20.5)
Paying attention to pressure points	63 (46.7)	41 (32.8)	18 (20.5)
Performing lab test for nutritional assessment	58 (46.7)	38 (31.1)	27 (22.1)
Providing vitamins and foods for patient	65 (53.3)	37 (30.3)	20 (16.4)
Monitoring protein and calories diet	56 (45.9)	49 (40.2)	17 (13.9)
Using special mattress prevent PU	54 (44.3)	50 (41.0)	18 (14.8)
Turning patient every 2 h	75 (61.5)	36 (29.5)	11 (9.0)
Using air bed for patients at high risk	52 (42.6)	51 (41.8)	19 (15.6)
Attending seminars for PU prevention	41 (33.6)	58 (47.5)	23 (18.9)
Giving advice for patient or care giver	85 (69.7)	26 (21.3)	11 (9.0)

#### Nurses’ perceived barriers for practicing PUP

Nurses were asked to indicate their agreement about the existence of specific barriers in the work environment. Among the nurses participated in the study, all of them had reported at least one of the following major challenges: heavy workload, inadequate staff, lack of universal guideline on prevention of pressure ulcer, inadequate coverage about PU during training, shortage of resources (equipment, facilities), uncooperative patients, job satisfaction in nursing, lack of time and lack of awareness.

Those who were involved in heavy workload were 2.594 (0.126–0.993) times more risky for pressure ulcer than their counterpart. Moreover, those who have knowledge were less risky (0.354 (0.126–0.993)) than their counterpart (Table 3).

**Table 3:** Frequency distribution of nurses’ perceived barriers to prevent pressure ulcer in public hospitals in central zone Tigray, 2018 (N = 122)

Variables	Nurses practice		COR (95% CI)	AOR (95% CI)
	Good (%)	Poor (%)		
Sex				
Male	8 (38.1)	46 (45.5)	1.359 (0.518–3.653)	
Female	13 (61.9)	55 (54.5)		
Age				
20–29	15 (17.6)	70 (82.4)	1.867 (0.33–10.551)	
30–39	4 (13.3)	26 (87.7)	2.60 (0.370–18.249)	
≥ 40	2 (28.6)	5 (71.4)	1.00	
Income				
Lowest	6 (32.5)	10 (62.5)	0.273 (0.081–0.919)	0.221 (0.058–0.840)
Medium	5 (14.3)	30 (85.7)	0.984 (0.309–3.135)	0.77 (0.224–2.70)
Highest	10 (14.1)	61 (85.9)	1.00	1.00
Year of experience				
1–4	5 (14.7)	29 (85.3)	1.933 (0.483–7.43)	
5–10	11 (16.2)	57 (83.8)	1.727 (0.520–5.737)	
≥ 10	5 (25)	15 (75)	1.00	
Educational level				
Diploma	6 (24)	19 (76)	1.727 (0.520–5.737)	
Degree	15 (15.5)	82 (84.5)	1.00	
Heavy workload				
Yes	13 (14.6)	76 (85.4)	1.871 (0.695–5.034)	2.594 (0.126–0.993)
No	8 (24.2)	25 (75.8)	1.00	1.00
Lack of time				
Yes	12 (17.9)	55 (82.1)	0.897 (0.347–2.316)	
No	9 (16.4)	46 (83.6)	1.00	
Job satisfaction				
Yes	10 (16.4)	51 (83.6)	1.122 (0.438–2.875)	
No	11 (18.0)	50 (82.0)	1.00	
Knowledge				
Adequate	10 (27.8)	26 (72.2)	*2.622 (0.999–6.887)*	*0.354 (0.126–0.993)*
Inadequate	11 (12.8)	75 (87.2)	1.00	1.00

Italics indicates significant association at p-value less than 0.5

### Discussion

The current findings showed that nurses who participated in the study had a good knowledge (82.75%) towards pressure ulcer prevention which is higher than the finding of the study in Alexandria Insurance Hospitals (< 70%) with similar study participants [20] as well as the finding in a study conducted in Bangladesh (57.79%) [13]. But, higher than the study conducted in among 3 public hospitals of AA Ethiopia (Black lion, St Paul’s, Ras Desta Damtew) [21] and across 6 public hospitals (Black Lion, Zawuditu Memorial, Alert, Yekatit, Tirunesh Beijing, and Menilik II) which revealed that, the overall nurses knowledge towards PUP was 61.2% and 63.85% respectively [22]. The finding was also higher than the study conducted in Gondar University Teaching Hospital which was at 54.4% [7]. These discrepancies may occur due to a different scoring method and a different sample size.

According to this study, the highest scores of nurses’ knowledge on PUP found in the educational program of pressure ulcers (79.5%) which was less than in Ugandan nurses (92.2%) [23]. conversely, there was a lower score of nurses’ knowledge of pressure ulcer prevention. the discrepancy may be due to lack of universal training or presence of other priorities as nurses reported lack of universal guideline on prevention of pressure ulcer and presence of other priorities than pressure ulcer which may have become one of their major barrier in PU prevention. Furthermore, 17.2% of the participants had good practice regarding PUP which is consistent with the study conducted in Nigeria (27.68%) [11]. But this figure is different from the study conducted in Gondar University Teaching Hospital [21] in which nearly half of nurses 48.4% have good practice towards PUP practice. This might be due to different evaluation tools and scoring system.

Among the practices that nurses always carry out, giving advice for the patient (69.7%), turning patients every 2 h (71.5%) and identifying PU contributing factors (59.0%) were the most practiced. Unlike this, a study conducted in Nigeria [11] showed that turning of patients every 2 h and using of special pillow to prevent PUP were the most practiced care for patients to prevent PUP. The difference between the healthcare setups of Ethiopia and Nigeria might explain the relative discrepant results in PUP practice.

The reason for the very low level of nurses’ practice could be supported by Moore and Prince and study in Uganda which means that the reported nurses’ barriers may be the reasons for very low level of PU prevention. This study showed that nurses’ practice almost reflected by nurses’ knowledge towards PU prevention.

The heavy workload and shortage of equipment were the most reported barriers to PUP practice. This report is similar to the study conducted in Uganda in which the majority of nurses report a lack of adequate staff as a barrier for implementing effective care practice related to PUP. Similarly, the studies conducted across 6 public hospitals in AA and Gondar University also cited heavy workload as the most reported barrier to practice PUP [7]. Studies have suggested that pressure ulcer development can be directly affected by the number of nurses and the time they spent at the bedside [24].

### Conclusion

- More than half of the nurses were found to have inadequate knowledge regarding PUP and
- Nurses’ levels of practices were found to be very poor concerning prevention of PU.

## Limitation of the study

The study was cross-sectional which didn’t indicate the precedence of outcome or exposure.

## Data Availability

The data sets used and analyzed during the current study available from the corresponding author on reasonable request.

## Abbreviations

AOR: Adjusted odd ratio
CI: Confidence interval
PU: Pressure ulcer
PUP: pressure ulcer prevention
SPSS: Statistical Package for Social Science
USA: United States of America
